# Equine cyathostomins: a review of biology, clinical significance and therapy

**DOI:** 10.1186/1756-3305-2-S2-S1

**Published:** 2009-09-25

**Authors:** Susan Corning

**Affiliations:** 1 Fort Dodge Animal Health Italy, Via G. Amendola, 8, 40121 Bologna, Italy

## Abstract

The small strongyles of horses, also known as cyathostomins, are considered the most prevalent and pathogenic parasites of horses today. The clinical syndrome of larval cyathostominosis which occurs as a result of mass emergence of inhibited stages has a high fatality rate despite the best standard of care given to affected horses. Management of the challenge level of cyathostomins to prevent the syndrome is preferable. Many different management programmes have been tried over the past two decades, with mixed success. Programmes have relied heavily on repeated use of anthelmintic treatments throughout the life of a horse. The widespread incidence of resistance to certain anthelmintics is reducing these options. An understanding of the biology of cyathostomins, risk factors for infection and appropriate strategic use of still effective anthelmintics is essential for the future management of this parasite group. This review highlights the necessity to use currently available anthelmintics that are appropriately suited to the biology of cyathostomins, and to maintain heir efficacy through an appropriate treatment strategy.

## 

Small strongyles (*Nematoda, Strongylida*) or "cyathostomins" have been reported from horses worldwide. They are highly prevalent in equine populations regardless of climatic or management differences, and seem equally at home in horses in the tropics as in temperate or cold climates [1-3]. Concerns have been raised in many aspects of managing cyathostomin challenges in horses, ranging from increasing prevalence, resistance to anthelmintic drugs and how to prevent and manage the clinical syndrome of larval cyathostominosis. There are more than 50 species of cyathostomins recognised [1,4] with some 10 species reported to be the most prevalent. They are today the most common and pathogenically significant parasite to affect horses around the world [3,5,6].

Although much has been written about the prevalence [3,5,7-13] and dangers of these nematodes, a brief review of their biology and clinical significance can be useful in understanding the need for developing appropriate and effective anthelmintic strategies to best protect horses from these highly pathogenic invaders.

## Life-cycle and epidemiology

Cyathostomins are commonly known as "small red-worms" due to the fact that they are usually less than 2.5 cm in length, and sometimes appear more red than white in colour. Like many other nematodes, cyathostomins have a direct lifecycle, with no intermediate host.

**Figure 1:**
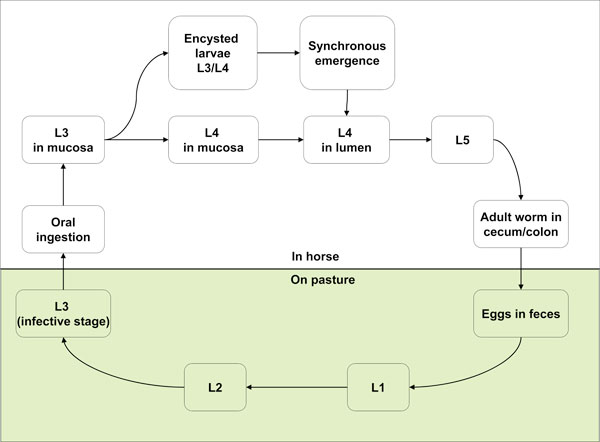
**Life cycle of cyathostomins**.

A schematic description of the life cycle of cyathostomins is presented in Figure 1. Cyathostomins enter the intestine at the third larval stage (L3) which has developed from eggs passed through the faeces onto pasture land. Once ingested by the horse, they continue their maturation and, in a "fast" life cycle, new eggs may be passed in the faeces onto the pasture within 5-6 weeks. The rate of development from the first larval stage (L1) to L3 stage is directly proportionate to temperature: in warm weather, eggs can hatch and yield infective L3 in as little as 3 days. Once they reach the L3 stage, they become surrounded by a protective membrane, and can survive well even in freezing conditions, which means that they have the ability to remain on the pasture for a prolonged period. In the case of experimental infections, the development time of infections could be influenced by chilling the larvae, or by administering a trickle infection rather than a single infection. In the case of a single infection, the mean pre-patent period was 53 days with a range of 48 to 62 days, while with trickle infection the mean pre-patent period was 65 days with a range of 60 to 77 days [14].

Moreover, cyathostomins differ from other worm species in that the maturation of the early third larval stage (EL3) might be arrested for a prolonged period of time. After ingestion, the L3 ex-sheath and invades the mucosa of the large intestine. Once inside, the larval stages protect themselves by becoming encysted. In fact, up to 90% of encysted cyathostomins may become "inhibited" in their EL3 stage of development [4], and can remain within the intestinal wall for periods ranging from about 4 months to as long as 2 years.

The season during which inhibition occurs varies dependent on climate. In temperate climates accumulation of larvae will occur during the grazing season, the larvae will encyst during the cooler months of the year, and may emerge en-masse as the weather warms up in spring. The reverse timing is seen in tropical climates, where the most likely timing for inhibition is during the stressful, hot summer months, with larval emergence in autumn [15].

Therefore, small strongyles have the ability to survive both on the pasture and inside the horse for very long periods of time, and effective, sustainable management and treatment programmes need to take into consideration the climatic conditions and lifecycle.

The important epidemiological risk factors for infection with cyathostomins have been identified as age, season and time since last deworming [16]. Interestingly, access to grazing and shared grazing with other horses were only weakly associated with cyathostominosis.

## Species prevalence

While the classification of small strongyle species of horses has been the subject of some discussion, there is general agreement that more than 50 species may be involved in parasitism of horses. Identification of species is usually performed on adult rather than larval stages, although in more recent times in vitro testing based on genotype has been developed [17].

Information is available on the most prevalent species of cyathostomins from a number of continents [3,5,7,9,18] and from differing climatic regions within continents. Often, cyathostomins were present in the majority of horses surveyed (>70% to 100%) with multiple species present in individual animals. The number of species ranged from a few to up to 26, but there was a remarkable similarity in the predominant species, regardless of geography. For example, *Cyathostomum catinatum, Cylicocyclus nassatus *and *Cylicostephanus longibursatus *were found to be amongst the most prevalent five species in France, Ukraine, US and Australia. *C. nassatus *was also common in Brazil. Other species that were widely recognized included *Cylicostephanus minutus, Cylicostephanus calicatus *and *Cyathostomum insigne*. Ambient temperature does not appear to be a significant factor in species distribution, as the mix of species reported from tropical and temperate climatic zones in Australia was very similar [3,9].

Little is understood about the relative pathogenicity of individual species, or what determines the balance of species in any mixed population. Some species are known to predominantly reside in the colon, while others seem to prefer the caecum [19]. Little is also known about individual species' lifecycles in terms of patency period, although it has been reported that those species residing in the caecum appear later in the faeces than species residing in the colon [20].

## Clinical significance

Small strongyles also have a remarkable ability to be pathogenic to the horse from the moment that they enter its gut. In common with other nematodes, large numbers of adult worms may cause clinical symptoms such as lethargy, sudden weight loss, debilitation, and diarrhoea. However, cyathostomin larval stages can cause even more serious problems. At the start of their invasion, the ex-sheathed L3 may cause serious damage to the intestinal mucosa. Once at the encysted stage, tens of thousands of encysted larvae may literally cover the mucosa wall, severely damaging it and greatly reducing nutritional metabolism [5]. The wall of the cyst protects the larva, and thus it may be unaffected by conventional dewormers.

But the most devastating damage can arise from the developed L4 stage when it emerges from the cyst and continues its development to the adult stage in the intestinal lumen. This usually occurs in the late winter or early spring, when enormous numbers of larvae emerge into the gut lumen *en masse*. This condition, known as "larval cyathostominosis", can severely damage the gut wall, with a resultant diarrhoea, potentially serious colic, and a mortality rate as high as 50% [21-29]. Granulomatous colitis has also been reported associated with small strongyle larvae [30].

Although young horses are most vulnerable [6], it is important to note that there is a lifelong susceptibility to cyathostomins, and that they can cause clinical disease in any age of horse during any season [31]. Because cyathostomins can be pathogenic both at penetration into and emergence from the large intestinal mucosa, it is essential that both incoming and encysted larvae are effectively eliminated. In the case of the encysted cyathostomins, their potentially long arrested development may be influenced by a larger larval challenge dose and how many grazing seasons have been experienced [31].

## Clinical and histopathological diagnosis

Since the clinical signs and clinical pathology associated with cyathostomins are not specific and are similar to a number of other conditions, diagnosis can be challenging. However, a rapid diagnosis is a necessity if appropriate treatments are to be administered with due speed, as this can be a condition with high mortality. Unfortunately, many times a diagnosis may only be arrived *post mortem *[32]. Affected horses may be of any age and present with any of the following signs: chronic diarrhoea, oedema, anorexia, dullness, acute weight loss pyrexia [24,33]. Similarly haematology is not diagnostic.

A typical clinical picture includes neutrophilia, hypoalbuminaemia, hyperglobulinaemia, especially beta-globulin, all are findings that are consistent with a protein-losing enteropathy. Low total serum protein has been reported, as well as slightly high total protein [33,34], possibly a result of dehydration. Diagnosis based on clinical and haematological findings includes a horse presenting in poor condition with diarrhoea, a serum albumin concentration of less than 20 g/L and a ratio of albumin:globulin of less than 0.7. Such a case is very likely to be infected with adult and L4 stages of cyathostomins [35]. There may be anaemia with or without eosinophilia and/or lymphocytosis [22] One useful diagnostic finding is the presence of large numbers of cyathostomin larvae in faeces, but their absence does not necessarily rule out cyathostomins as a cause of the clinical condition [21,35].

Necropsy findings show inflammation of colon and/or caecum, in the acute stage there is marked mucosal hyperaemia, haemorrhage, congestion, ulceration or necrosis. In more chronic cases there may be only some mucosal thickening due to oedema, and irregular areas of congestion. Numerous small strongyle larvae can be seen in the mucosa on careful inspection. Transillumination using a powerful lights source from the serosal surface will aid their detection [21].

On histopathology, a cellular and inflammatory response will be seen including mixed populations of mononuclear cells, eosinophils and epithelial cells. The response may focus around the larvae in the submucosa, or may be more diffuse involving the mucosal lamina propria as well as the submucosa [21].

## Current control measures

Without a doubt, managing the level of small strongyle challenge, particularly the avoidance of the larval cyathostominosis syndrome, is the preferred path. Treatment of clinical cases can be protracted, difficult and unrewarding, with mortality rates of 40-70% reported even with aggressive treatment.

The plethora of publications describing possible control measures are a testament that control is difficult, that there is no one programme that can be used under all circumstances but that control practices need to be tailored to individual horse farms and yards. Control measures inevitably involve the use of anthelmintics, however, the ever increasing prevalence of resistance adds another level of difficulty to designing appropriate control programmes.

It is therefore advisable to use an anthelmintic with a high efficacy that will substantially reduce larval pasture burdens, and one which is proven to be effective against encysted cyathostomins. It is only with the best weapons and an effective strategy that the challenge of combating these highly pathogenic invaders can be achieved.

Over the past 20 years, management of cyathostomins has relied heavily on the repeated use of anthelmintic drugs. Various drug treatment regimens have been recommended, often recommending set interval treatments without regard to drug properties, age of horses [36,37] or epidemiology of the cyathostomins, and attempts have been made to address better control strategies [38-43].

The objectives of effective control programmes should address measures to reduce the numbers of infective larvae on pastures and to reduce the number of anthelmintic treatments required to achieve this egg reduction as a means of delaying or avoiding drug resistance in the cyathostomin population [44].

There are three available drug classes for cyathostomin control in horses, the benzimidazoles such as fenbendazole and oxfendazole, the tetrahydropyrimidines which are the pyrantel salts, and the macrocyclic lactones (ML), ivermectin and moxidectin. All of these drugs have differing levels of efficacy, duration of activity and spectrum of stages of cyathostomins they control. The ML class of drugs has become ever more widely used due to their potency, spectrum of activity, relative safety, and as yet few reports of resistance.

In the case of fenbendazole, the recommended dose of 5 mg/Kg liveweight will control sensitive strains of adult and developing larval stages of small strongyles. For control of inhibited stages a daily dose of 10 mg/Kg liveweight for 5 consecutive days is recommended. Fenbendazole resistance has been recognised as being widespread in all major horse populations surveyed and use of this compound at either dosage regimen should be avoided where resistance occurs [45-51].

Treatment regimens with pyrantel salts have varied, and include recommendations for monthly treatments, or even daily administration which was adopted in horse operation the US for many years, although this programme did not find favour in other geographies.

Pyrantel salts are not effective against inhibited stages of small strongyles but will remove sensitive strains of adults. Resistance to pyrantel salts has been identified both in Europe and the US, but does not appear to be as widespread as resistance to benzimidazoles [52-57].

As a general caution, unless sensitivity has been demonstrated by a faecal egg count reduction test, use of benzimidazole or pyrantel based anthelmintics carries the risk that treatment will be ineffective [58-61].

The two compounds within the macrocyclic lactone group need to be considered separately due to significant differences in potency and spectrum. The first available ML for horse, ivermectin, is highly potent against adult stages, luminal larval stages and developing stages of larvae in mucosa, but has variable and low efficacy against inhibited stages, even when elevated doses (5×) are administered [62-64].

Moxidectin, in addition to having high efficacy against all cyathostomin stages given as a single dose at a rate of 0.4 mg/Kg liveweight [62-66] also provides persistent activity against re-infection by small strongyles [67], resulting in a long egg re-appearance interval. The required re-treatment interval with moxidectin is longer than that for other anthelmintics allowing less frequent treatment and less selection for resistance [44,57,68-75].

The effects of removal of luminal stages of parasites on emergence of inhibited stages, whether by anthelmintic treatment or natural expulsion, must also be taken into account when designing new approaches to cyathostomin control programmes [76]. Another factor of importance to clinicians is the consequence of killing the inhibited stages, as it has been reported that the die-off of these stages following fenbendazole treatment results in severe inflammation of the mucosa of the colon. In the same study, inflammation was not seen subsequent to the elimination of these stages using moxidectin [77].

## Conclusion

We are fortunate that the last two decades have seen a large body of research, information and understanding of the complex issues surrounding the life cycle, clinical significance and control of small strongyles in horses. We have the opportunity to apply this knowledge to develop better control programmes than have been implemented in the past.

Monahan summed the situation well by stating in 2000: "Rote memorization of treatment schedules and anti-parasitic drugs without understanding the biology of the worms to be controlled concedes any intellectual advantage to the worms" [44].

## Competing interests

The author declares no conflicting interests in the preparation or content of this review.
